# How Does Anodal Transcranial Direct Current Stimulation of the Pain Neuromatrix Affect Brain Excitability and Pain Perception? A Randomised, Double-Blind, Sham-Control Study

**DOI:** 10.1371/journal.pone.0118340

**Published:** 2015-03-04

**Authors:** Bita Vaseghi, Maryam Zoghi, Shapour Jaberzadeh

**Affiliations:** 1 Department of Physiotherapy, School of Primary Health Care, Faculty of Medicine, Nursing and Health Sciences, Monash University, Melbourne, VIC, Australia; 2 Department of Medicine, Royal Melbourne Hospital, The University of Melbourne, Melbourne, VIC, Australia; University of Regensburg, GERMANY

## Abstract

**Background:**

Integration of information between multiple cortical regions of the pain neuromatrix is thought to underpin pain modulation. Although altered processing in the primary motor (M1) and sensory (S1) cortices is implicated in separate studies, the simultaneous changes in and the relationship between these regions are unknown yet. The primary aim was to assess the effects of anodal transcranial direct current stimulation (a-tDCS) over superficial regions of the pain neuromatrix on M1 and S1 excitability. The secondary aim was to investigate how M1 and S1 excitability changes affect sensory (STh) and pain thresholds (PTh).

**Methods:**

Twelve healthy participants received 20 min a-tDCS under five different conditions including a-tDCS of M1, a-tDCS of S1, a-tDCS of DLPFC, sham a-tDCS, and no-tDCS. Excitability of dominant M1 and S1 were measured before, immediately, and 30 minutes after intervention respectively. Moreover, STh and PTh to peripheral electrical and mechanical stimulation were evaluated. All outcome measures were assessed at three time-points of measurement by a blind rater.

**Results:**

A-tDCS of M1 and dorsolateral prefrontal cortex (DLPFC) significantly increased brain excitability in M1 (p < 0.05) for at least 30 min. Following application of a-tDCS over the S1, the amplitude of the N20-P25 component of SEPs increased immediately after the stimulation (p < 0.05), whilst M1 stimulation decreased it. Compared to baseline values, significant STh and PTh increase was observed after a-tDCS of all three stimulated areas. Except in M1 stimulation, there was significant PTh difference between a-tDCS and sham tDCS.

**Conclusion:**

a-tDCS of M1 is the best spots to enhance brain excitability than a-tDCS of S1 and DLPFC. Surprisingly, a-tDCS of M1 and S1 has diverse effects on S1 and M1 excitability. A-tDCS of M1, S1, and DLPFC increased STh and PTh levels. Given the placebo effects of a-tDCS of M1 in pain perception, our results should be interpreted with caution, particularly with respect to the behavioural aspects of pain modulation.

**Trial Registration:**

Australian New Zealand Clinical Trials, ACTRN12614000817640, http://www.anzctr.org.au/.

## Introduction

Pain is a multidimensional phenomenon with sensory-discriminative, affective-motivational, motor and autonomic components [1–5]. Primary (S1) and secondary (S2) somatosensory cortices, the thalamus, and posterior part of the insula collectively called lateral pain system which are responsible for sensory-discrimination of pain [6]. In contrast, the anterior cingulate cortex (ACC) and anterior part of the insula have been involved in affective-motivation processing of pain, which is referred to as the medial pain system [4, 7–9]. Cognitive aspects of pain is related to dorsolateral prefrontal cortex (DLPFC) [1]. Recent studies have shown that the motor cortex is also involved in pain modulation [10–16]. Some other areas of the brain including the pre-acuectal grey matter (PAG) system and nucleus cuneiformis also play a major role in modulation of pain [6]. Involvement of these areas of brain in pain processing occurs in a large distributed neural network called pain neuromatrix (PNM) [17]. Some parts of the PNM such as S1, M1, and DLPFC are superficial, and some others such as the thalamus, insula, and anterior cingulate cortex are deep structures [1, 4].

A growing body of evidence indicates co-activation of S1, M1 and DLPFC during pain processing [7, 18, 19], which may provide evidence for functional connectivity between these cortical sites. The connectivity between M1 and S1 has already been established by a number of studies. Matsunaga et al. showed that anodal transcranial direct current stimulation (a-tDCS) over the M1 can induce a long-lasting increase in the size of ipsilateral cortical components of sensory evoked potentials (SEPs) [20]. This connectivity was also studied by Schabrun et al. looking at the pain-induced changes in S1 and M1 excitability. They showed that the S1 excitability was reduced during and after pain, while M1 excitability was suppressed only after the resolved pain [21]. Furthermore, positron emission tomography (PET) studies also indicated mixed results which indicate that this relationship is not very straight forward. They showed that pain-induced S1 activity may coincide with increased [22, 23], decreased [24], or unchanged [25] M1 activity. Therefore, there is no consensus on direction of M1 activation in response to pain induced changes in S1. The relationship for DLPFC and M1 is more straightforward. Despite the direct effects of DLPFC on the frontal-parietal network and subgenual cortex [26], literature indicates that increased DLPFC activity coincides with increased M1 activity and modulation of the medial pain system [27, 28].

Recent investigations have also demonstrated that the excitability changes in superficial areas of PNM induces changes in some key variables operationalizing pain, including sensory threshold (STh) and pain threshold (PTh) [29–31]. It is reported that increasing the excitability of M1 and/or S1 results in different STh/PTh responses [32, 33]. Based on the results of a recent systematic review, there is a site-specific effect in STh/PTh modulation following an increase in the excitability of M1/S1 in healthy individuals and M1/DLPFC in patients with chronic pain [9]. Unlike M1 and S1, the role of DLPFC on STh/PTh has not been investigated. The closest study in this regard is a recently published systematic review by O’Connell et al. (2014) which indicated that excitability modulation following application of repeated transcranial magnetic stimulation (rTMS), tDCS, or cerebral electrotherapy stimulation (CES) over the DLPFC has no effect on the pain level in patients with chronic pain [34]. The above studies could be categorised in two groups: first, the induced changes in a single PNM site is followed by measurement of excitability and STh/PTh changes in another site. Second, pain induced temporal association between the changes in activity of PNM sites are studied. To the best of our knowledge, there is no study in the literature to collectively investigate the effects of changes in one of these three superficial sites of PNM on the other two sites.

A-tDCS is a powerful non-invasive neuromodulatory technique which could be used to study the functional connectivity [35–37]. Therefore, in the present study, the primary aim is to simultaneously measure the level of M1 and S1 excitability following a-tDCS of M1, S1, and DLPFC to investigate the functional connectivities between these sites in healthy individuals. The secondary aim was to investigate how M1 or S1 excitability modulation affects STh and PTh. We also aimed to investigate the placebo effects of a-tDCS on modulation of M1, S1 excitability and STh and PTh. Indeed, the results of this pilot study generate further hypotheses relating to complex mechanisms of different brain stimulation localisations on sensory/pain thresholds.

## Materials and Methods

### Study design

We conducted a single-center, doubled-blinded, randomized, sham-controlled crossover study to determine the site-specific effect of a single session of a-tDCS on M1 and S1 excitability and STh and PTh in healthy volunteers. This study conformed to the ethical standards of the Declaration of Helsinki and was approved by the institutional ethics committee at Monash University, Clayton, Australia (S1 Ethics). Considering the WHO definition, it was impossible to publish the current study as an “Original Article”. As a result, it was registered as a clinical trial on the Australian New Zealand Clinical Trial (registry number: ACTRN12614000817640, http://www.anzctr.org.au/) after enrolment of all participants. The authors confirm that all ongoing and related trials for tDCS studies are registered.

### Participants

Between October and December of 2013, we conducted 60 experiments on 12 healthy volunteers (four men and eight women, all Monash University students) with a mean age of 23.6±5.3 years (age range 20–34). All were right-handers as determined by the Edinburgh Handedness Inventory (10-item version, mean laterality quotient = 87.9±10.5) [38]. Eligibility criteria were: age between 18 and 35 years, no clinically significant or unstable medical, neuropsychiatric, or chronic pain disorder, no history of substance abuse or dependence, no use of central nervous system-effective medication, no history of brain surgery, tumour, or intracranial metal implantation. All participants were interviewed and examined by a physician prior to enrolment in the study and provided written, informed consent. The protocol for this trial and CONSORT checklist are available; see S1 CONSORT Checklist and S1 Protocol.

### Experimental procedures

**Fig 1:**
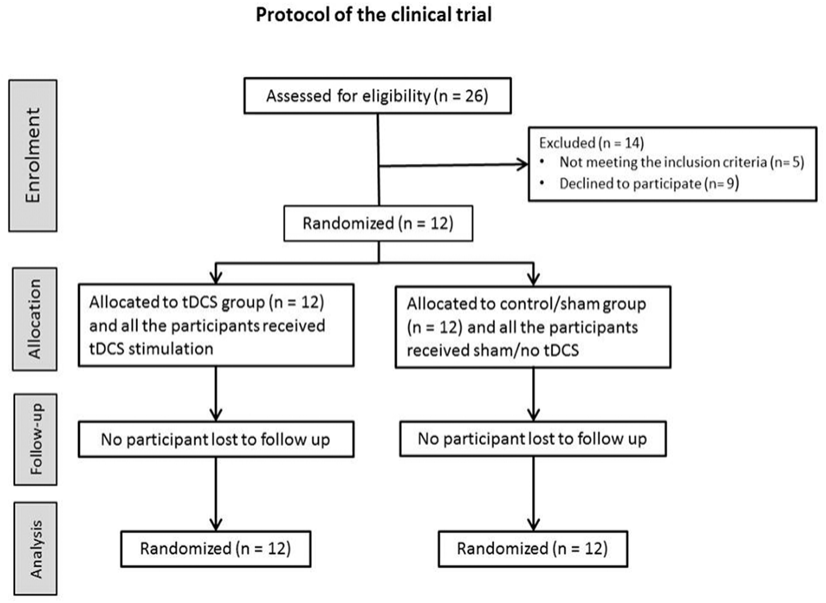
Flow diagram of the progress through the phases (Enrolment, Allocation, Follow-up, and Analysis) of the randomized clinical trial of transcranial direct current stimulation (tDCS) and sham/control groups.

The healthy participants received intervention with tDCS under each of five different conditions in a random order: a-tDCS of M1, a-tDCS of S1, a-tDCS of DLPFC, sham a-tDCS, and no-tDCS. The experimental sessions were separated by at least 72 hours to avoid interference or carry-over effects of tDCS, and completed at the same time of day (mornings or early afternoon) to avoid diurnal variation. The duration of tDCS application was 20 minutes in all experiments. MEPs, SEPs, STh, PTh to peripheral electrical stimulation, and PTh to pressure stimulation (P_p_Th) were assessed before (T_pre_), immediately after (T_0_) and 30 minutes (T_30_) after each intervention. Participants were blinded to the condition of tDCS (sham or active). The progress of the clinical trial through various phases (Enrollment, Allocation, Follow-up, and Analysis) is shown in Fig. 1. Two researchers (assessor of outcome measures and tDCS administrator) were involved in the current study; the assessor who measured SEP, MEP, STh, PTh, and P_p_Th and took part in data analysis, was blinded to all experimental conditions. The tDCS administrator, who was responsible for delivering the tDCS was not blinded to the tDCS condition.

### A-tDCS of superficial regions of PNM

Anodal tDCS was administered through an active saline-soaked surface sponge electrode (2×1.5cm) over the target area and a reference electrode (3×4cm over the right contralateral supraorbital area [35]. The larger size for the reference electrode decreases current density (CD) and reduces side effects under the indifferent electrode with more focused density under the anode [39]. The tDCS stimulator (Intelec Advanced Therapy System, Chattanooga, USA) was programmed to deliver 0.3mA direct current for 20 minutes, with 10 seconds of linear fade in and fade out. The electrodes were fixed with two horizontal and perpendicular straps.

Current intensity was set at 0.3mA which enabled us to considerably decrease the size of the electrodes [40]. In all experiments, the CD was kept at 0.1 mA/cm^2^ which is in a safe range with limited side effects [35, 41, 42]. There is evidence for superiority of this intensity in induction of corticospinal excitability [41, 43–45]. The small size of the anode electrode authorized highly focused stimulation of M1 and S1 [46].

**Fig 2:**
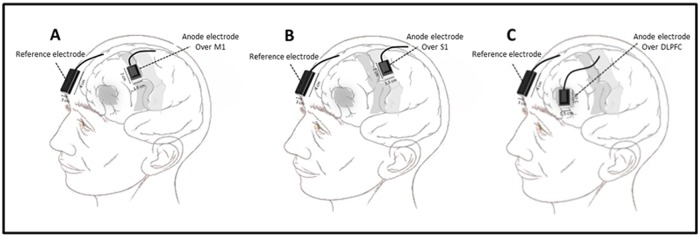
Schematic illustration of electrode montage. Stimulation of primary motor cortex (M1), primary sensory cortex (S1), and dorsolateral prefrontal cortex (DLPFC), the anode electrode was positioned on C3 (A), C′3 (B), and F3 (C) consecutively. The reference electrode was placed over the contralateral supraorbital area in all conditions.

For anodal stimulation of M1 and S1, the anode electrode was placed over C3 and C′3 (2cm backward relative to C3) based on the 10–20 system. For anodal stimulation of DLPFC, the anode electrode was placed over F3 (Fig. 2). The reference electrode (cathode) was conventionally placed over the contralateral supraorbital area with an assumption of negligible or zero neuromodulatory effects on the subgenual cortex. We kept the size of this electrode four times larger than the active anode (the density was four times less) to minimize neuromodulatory effects under the cathode, but in reality, the subgenual cortex may be affected even by this very low density of currents under the cathode [47–49]. In the sham condition, the electrodes were placed in the same positions as for anodal M1 or S1 stimulation randomly, but the stimulator was turned off after 30 seconds of stimulation. For the no-tDCS session, participants were asked to sit in a podiatry chair during the 20-minute intervention time although no electrode was placed on PNM regions. All pre and post evaluations were identical to those in other conditions.

### Measurement of side effects

To record side effects or adverse effects resulting from stimulation, all participants were asked to complete questionnaires both during and after all experimental conditions. The questionnaire contained rating scales for the presence and severity of side effects such as itching, tingling, burning sensations under electrodes [50, 51] as well as adverse effects including headache and pain during and after stimulation. All participants rated the unpleasantness of any scalp sensation using numeric analogue scales (NAS) (e.g., 0 = no tingling to 10 = worst tingling imaginable).

### M1 excitability measurement

Participants were seated upright in an adjustable podiatry chair, with the forearm pronated and the wrist joint in neutral position resting on the armrest. Single-pulse magnetic stimuli were delivered using a Magstim 2002 (Magstim Company Limited, Whiteland, Wales, UK) stimulator with a flat 70mm figure-of-eight standard magnetic coil (peak magnitude field 2.2T). The vertex (Cz) point was measured and marked to be used as a reference [52]. The magnetic coil was placed over the left hemisphere (cortex), contralateral to the target muscle. The orientation of the coil was set at an angle 45° to the midline and tangential to the scalp such that the induced current flowed in a posterior-anterior direction in the brain. A scalp site optimal for evoking an MEP in the first dorsi interosseous (FDI) muscle of the right hand was found and marked as a reference. The coil position and orientation were constantly checked during the experiment to ensure that no changes occurred.

MEP resting threshold (RT) was tested in steps of 2% maximum stimulator output [35], and defined as the lowest intensity for which five of ten successive MEPs exceed 50μV (rest) peak-to-peak amplitude [53–55]. For all further MEP measurements, the TMS intensity was set at 120% of each individual’s RT. Fifteen stimuli were elicited to assess corticospinal excitability of M1 at each time point. The stimulus intensity remained constant throughout the study session for each participant.

Surface EMG was recorded from the right FDI muscle using bipolar Ag/AgCl disposable surface electrodes with an inter electrode distance of 3cm (measured from the center of the electrodes). To ensure good surface contact and reduce skin resistance, a standard skin preparation procedure of cleaning and abrading was performed for each electrode site [52, 56]. The location of FDI was determined based on anatomical landmarks [57] and also observation of muscle response in the testing position (abduction of index finger toward the thumb) [58]. The accuracy of EMG electrode placement was verified by asking the participant to contract the muscle of interest while the investigator monitored online EMG activity. A ground electrode was placed ipsilaterally on the styloid process of the ulnar bone [59, 60]. The electrodes were secured by hypoallergenic tape (Micropore, USA). All raw EMG signals were band pass filtered (10–1000Hz), amplified (61000) and sampled at 2000Hz and collected on a PC running commercially available software (ChartTM software, ADinstrument, Australia) via a laboratory analogue-digital interface (The PowerLab 8/30, ADinstrument, Australia). Peak-to-peak MEP amplitude was detected and measured automatically using a custom-designed macro in Powerlab 8/30 software after each magnetic stimulus.

### S1 excitability measurement

SEP were recorded following electrical stimulation of the right median nerve at the wrist level at 2Hz with pulse width of 0.2ms [61]. The intensity of stimulation was fixed at the motor threshold [61]. At this stimulus intensity, SEPs were recorded from S1 using electroencephalography (EEG) electrodes. One electrode was located over the C′3 (2cm behind C3) and the reference electrode was placed over the mid-frontal (Fz) position [20, 62]. The electrical potentials were recorded in epochs from 0 to 200ms after the stimulus. A total of 500 stimulus-related epochs were recorded. Peak-to-peak amplitude of N20-P25 responses generated in S1 were measured and compared before and after tDCS stimulation in different areas of PNM [63].

### Measurement of STh and PTh

All evaluations were performed at T_pre_, T_0_, and T_30_ by a blinded rater. The primary outcomes were STh and PTh to electrical stimulation. Electrical stimulation was applied by a pen electrode (model: 2762CC, Chattanooga, USA) to the right median nerve (pulse duration: 200μs) at wrist level. Current supply started at 0mA and was increased in steps of 0.1mA until the participant reported sensation and pain. The intensity of current at which perception of the electrical stimulus was first reported was taken as the STh; the intensity of current at which participants first reported pain was taken as the PTh and then averaged for analysis.

### Measurement of P_p_Th

Pressure was induced using a pressure algometer (model: FDX 50, Wagner, USA; capacity: 50× 0.05Ibf, accuracy: ±0.3% of full scale) with a flat circular metal probe dressed in a plastic cover. Force was displayed digitally in increments of 0.1N. The algometer was mounted vertically. For each measurement the algometer was calibrated to enable force to be applied at a controlled and steady rate. P_p_Th was defined as the amount of force required to elicit a sensation of pain distinct from pressure or discomfort [64]. The P_p_Th measurement point was marked in the middle of the belly of the FDI muscle [65].

Participants were instructed in the application of the algometer and given a demonstration. They then underwent two practice measurements on their non-dominant side. Participants were asked to say “stop” immediately when a discernible sensation of pain, distinct from pressure of discomfort, was felt; at this point, the experimenter retracted the algometer [66, 67]. The digital display continued to show the value of pressure applied at the moment the algometer was retracted. The algometer was applied perpendicularly to the skin and lowered at a rate of a rate of approximately 5N/s until P_p_Th was reached [64], as detected by participants’ verbal report. At each time point, three P_p_Ths were measured approximately 10–15s apart and then averaged for analysis.

### Data analysis

The data were analyzed, blinded to experimental conditions. The post-intervention means were normalized to intra-individually and are given as ratios of the baseline [54]. Using one-way repeated measures ANOVA at T_pre_ of all conditions, we sought to detect any carry-over effect at the starting point of each session.

A two-way repeated measures ANOVA was used to assess the effects of two independent variables, experimental conditions (a-tDCS of S1, M1, DLPFC, sham, and no-tDCS) and time points (T_pre_, T_0_, and T_30_), on MEPs, SEPs, STh, PTh and P_p_Th. Mauchly’s test was used to assess the validity of the sphericity assumption for repeated measures ANOVA; it requires that the variances for each set of difference scores be equal. Greenhouse-Geisser corrected significance values were used when sphericity was lacking [68]. Additionally, to test whether the baseline value of each stimulation site differed significantly from post-intervention time points (T_0_, T_30_), a paired-sample t-test was applied.

A significance level of P = 0.05 was adopted for all comparisons. A post-hoc test (Bonferroni) was performed where indicated. Means are reported ±SE. Statistical analyses were performed using SPSS software version 22.

## Results

### Comparison of baseline values

One-way repeated measure ANOVA showed that baseline values of dependent variables (peak-to-peak amplitude of MEPs and of N20-P25 of SEPs) remained unchanged in multiple sessions of assessment for all experimental conditions. This indicates that the wash-out period was adequate and refuses any possibility of carry-over effects from previous interventions on same participants.

### The effects of a-tDCS of M1, S1, and DLPFC on M1 excitability

**Fig 3:**
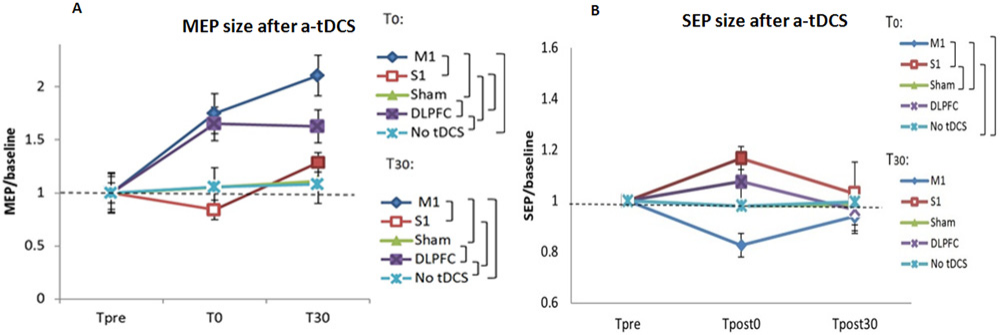
The effects of a-tDCS over different stimulation sites on MEP and SEP sizes. The peak-to-peak amplitude of MEPs (A), and the peak-to-peak amplitude of N20-P25 of SEPs (B) are illustrated following a-tDCS of primary motor cortex (M1), primary sensory cortex (S1), dorsolateral prefrontal cortex (DLPFC), and sham a-tDCS over time. Filled symbols indicate significant deviation of the post transcranial stimulation MEP and SEP amplitudes relative to the baseline; the brackets show significant differences between different testing conditions. Data are reported as mean ± SEM.

The two-way repeated measures ANOVA revealed significant main effects for the stimulation site, time, and site × time variables (Table 1). Post hoc comparisons showed significant difference between M1/S1, DLPFC/S1, M1/Sham or no-tDCS, DLPFC/Sham or no-tDCS, and S1/no-tDCS at both T_0_ and T_30_ (Fig. 3). Based on the results, there was no significant difference between M1-DLPFC and S1-sham at T_0_ or T_30_. No significant difference was found between sham and no-tDCS conditions. A two-tailed, paired sample t-test with an alpha level of 0.01 was used to compare baseline values with T_0_ and T_30_ in each experimental condition. The results indicated that a-tDCS of both M1 and DLPFC increased the size of MEPs at T_0_ and T_30_ significantly. Significant difference was observed between T_0_-T_30_ but not T_pre_-T_0_ or T_pre_-T_30_ following a-tDCS of S1 (Table 2).

**Table 1:** ANOVA results for the effects of a-tDCS on the MEP and SEP sizes and the level of STh, PTh, and P_p_Th.

		*df*	F-value	P-value
**MEP size**	**Stimulation site**	4	12.8	0.001
	**Time**	2	29.2	0.0008
	**Stimulation site× Time**	8	9.1	0.0001
	**MEP sizes at T_0_**	4	12.4	0.0008
	**MEP sizes at T_30_**	4	10.1	0.002
**SEP Size**	**Stimulation site**	4	4.3	0.005
	**Time**	2	0.37	0.03
	**Stimulation site× Time**	8	2.4	0.02
	**SEP sizes at T_0_**	4	5.8	0.001
	**SEP sizes at T_30_**	4	0.19	0.90
**STh**	**Stimulation site**	4	4.3	0.005
	**Time**	2	9.5	0.009
	**Stimulation site× Time**	8	3.6	0.001
	**STh sizes at T_0_**	4	2.8	0.35
	**STh sizes at T_30_**	4	6.0	0.001
**PTh**	**Stimulation site**	4	3.6	0.01
	**Time**	2	20.6	0.0008
	**Stimulation site× Time**	8	2.5	0.01
	**PTh sizes at T_0_**	4	1.8	0.17
	**PTh sizes at T_30_**	4	5.1	0.009
**P_p_Th**	**Stimulation site**	4	3.0	0.02
	**Time**	2	29.6	0.0009
	**Stimulation site× Time**	8	2.8	0.009
	**P_p_Th sizes at T_0_**	4	2.9	0.13
	**P_p_Th sizes at T_30_**	4	27.8	0.0009

**Table 2:** The effects of different experimental conditions on the size of MEPs/SEPs and the level of STh, PTh, P_p_Th.

		MEP	SEP	STh	PTh	P_p_Th
**M_1_ tDCS**	**T_0_**	1.74±0.11	0.82±0.04	1.49 ± 0.12	1.27 ± 0.09	1.18 ± 0.07
	**T_30_**	2.11±0.13	0.94±0.06	1.59 ± 0.12	1.45 ± 0.11	1.34 ± 0.08
	**P-Value (T_pre_-T_0_)**	0.002	0.003	0.003	0.012	0.02
	**P-Value (T_pre_-T_30_)**	0.002	0.40	0.004	0.002	0.001
	**P-Value (T_0_-T_30_)**	0.01	0.08	0.12	0.09	0.001
**S_1_ tDCS**	**T_0_**	0.84±0.09	1.16±0.04	1.87 ± 0.51	1.37 ± 0.11	1.63 ± 0.28
	**T_30_**	1.3±0.15	1.03±0.12	1.98 ± 0.37	1.49 ± 0.09	1.56 ± 0.25
	**P-Value (T_pre_-T_0_)**	0.11	0.004	0.105	0.009	0.04
	**P-Value (T_pre_-T_30_)**	0.08	0.81	0.025	0.000	0.01
	**P-Value (T_0_-T_30_)**	0.005	0.09	0.48	0.107	0.17
**DLPFC tDCS**	**T_0_**	1.65±0.15	1.07±0.09	1.60 ± 0.2	1.25 ± 0.12	1.05 ±. 06
	**T_30_**	1.62±0.07	0.96±0.08	1.75 ± 0.16	1.37 ± 0.09	1.22 ± 0.12
	**P-Value (T_pre_-T_0_)**	0.002	0.44	0.01	0.07	0.41
	**P-Value (T_pre_-T_30_)**	.000	0.63	0.001	0.002	0.11
	**P-Value (T_0_-T_30_)**	0.87	0.09	0.021	0.38	0.12
**Sham tDCS**	**T_0_**	1.05±0.05	0.97±0.01	1.08 ± 0.08	1.19 ± 0.16	1.28 ± 0.06
	**T_30_**	1.1±0.02	0.98±0.02	1.14 ± 0.30	1.19 ± 0.11	1.29 ± 0.07
	**P-Value (T_pre_-T_0_)**	0.06	0.23	0.30	0.26	0.07
	**P-Value (T_pre_-T_30_)**	0.053	0.45	0.14	0.09	0.03
	**P-Value (T_0_-T_30_)**	0.11	0.77	0.35	0.98	0.90
**No-tDCS**	**T_0_**	1.05±0.06	0.98±0.03	0.99 ± 0.04	0.97 ± 0.01	1.01 ± 0.03
	**T_30_**	1.08±0.02	0.99±0.01	1.03 ± 0.03	1.00 ± 0.02	0.99 ± 0.02
	**P-Value (T_pre_-T_0_)**	0.09	0.11	0.96	0.051	0.67
	**P-Value (T_pre_-T_30_)**	0.07	0.83	0.32	0.059	0.93
	**P-Value (T_0_-T_30_)**	0.12	0.17	0.14	0.15	0.60

The effect of a-tDCS of M1, S1, DLPFC, sham, and no condition on MEPs, SEPs,sensory (STh) and pain (PTh), and pressure pain threshold (P_p_Th) changes are illustrated at T0 and T30. T0 and T30 rows show Mean ± SE changes compared to baseline values. As the mean values normalized to baseline, the mean and post-intervention values are given as ratios of the baseline, the value of T_pre_ is considered as 1.

### The effects of a-tDCS of M1, S1, and DLPFC on S1 excitability

The results of two-way repeated measures ANOVA showed significant effects for the site of stimulation, time, and site×time variables (Table 1). Post hoc comparisons indicated that there was a significant difference between M1-S1, M1-sham/no-tDCS, and S1-sham/no-tDCS at T_0_. No significant change was found in the amplitude of N_20_-P_25_ 30 minutes after a-tDCS. The results also showed that there was no difference between sham and no-tDCS. Paired sample t-tests indicated that there was a significant difference in the size of SEPs at T_pre_ and T_0_ following a-tDCS of M1 and S1 (Fig. 3). No significant difference was found between SEP sizes at T_pre_ and T_30_ (Table 2).

### The effects of a-tDCS of M1, S1, and DLPFC on STh

**Fig 4:**
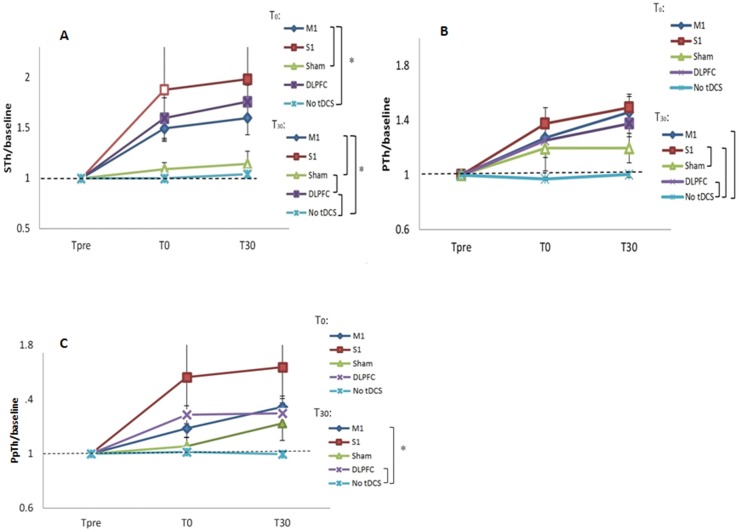
The effects of a-tDCS over different stimulation sites on sensory and pain threshold. Sensory threshold (STh) (A), pain threshold (PTh) (B), and pressure pain threshold (P_p_Th) (C) changes are illustrated following a-tDCS of primary motor cortex (M1), primary sensory cortex (S1), dorsolateral prefrontal cortex (DLPFC), and sham a-tDCS over time. Filled symbols indicate significant deviation of the post transcranial stimulation STh, PTh, P_p_Th relative to the baseline; the brackets show significant differences between different testing conditions. Data are reported as mean±SEM.

STh analysis showed significant main effects of stimulation site, time, and site × time (Table 1). As displayed in Fig. 3, there was no significant difference between M1, S1, and DLPFC at T_0_ and T_30_. The result indicated a significant difference between M1/sham or no-tDCS at T_0_ and T_30_, and DLPFC/sham or no-tDCS at T_30_. With regard to the STh level after a-tDCS of superficial regions of the PNM at each time point No significant STh changes were found between sham and no-tDCS conditions (Fig. 4). Furthermore, comparing baseline values, the results indicated that STh were significantly increased after a-tDCS of M1 and DLPF at both T_0_ and T_30_. Paired t-test results also showed that there is a significant STh increase after a-tDCS of S1 at T_30_ but not T_0_ (Table 2).

### The effects of a-tDCS of M1, S1, and DLPFC on PTh

The repeated measures ANOVA revealed significant main effects of the stimulation site, time, and stimulation site×time on PTh following a-tDCS of superficial areas of the PNM (Table 1). As shown by the post hoc tests, there was no significant difference between experimental conditions at T_0_. Significant differences were found between S1/sham or no-tDCS, M1/no-tDCS, DLPFC/no-tDCS, while there was no significant difference in PTh between sham and a-tDCS of M1 and DLPFC. The results also revealed no significant difference between sham and no-tDCS conditions (Fig. 4). Comparing baseline PTh with the values at T_0_ and T_30_ indicated that PTh increased immediately and 30 minutes after a-tDCS of S1. PTh increase was also found after a-tDCS of DLPFC and M1 at T_30_ but not T_0_ (P< 0.01) (Table 2).

### The effects of a-tDCS of M1, S1, and DLPFC on P_p_Th

A two-way repeated measures ANOVA was used to compare the effects of five different conditions on brain excitability at three time points. The results revealed significant main effects of time, stimulation site, and stimulation × time interaction. Post hoc comparison indicated that there were no significant changes at any sites of stimulation at T_0_. The results also showed significant differences in P_p_Th between M1/no-tDCS and DLPFC/no-tDCS conditions. No significant difference between sham and no-tDCS conditions was detected (Fig. 4). Compared to baseline values, pairwise comparison showed significant P_p_Th changes following a-tDCS of M1 at T_30_ (Table 2).

### Safety and side effects of a-tDCS

All participants tolerated the applied currents in different conditions very well and there was no interruption of experimental procedures due to adverse or side effects of the applied currents. Table 3 summarizes the means ± SEM for reported side effects under the anode and cathode for each of the experimental sessions. Itching is a side effect of a-tDCS, which was experienced by all participants in active and sham sessions. There were no side effects reported by participants after the end of stimulation. No reports of burning sensations, headaches, or pain were recorded during or after stimulation.

**Table 3:** Numeric sensation score by participants during experimental conditions.

		Anode electrode				Reference electrode			
		M1	S1	DLPFC	Sham	M1	S1	DLPFC	Sham
**Tingling sensation**	Beginning	4.6 ± 0.28	5.1 ± 0.42	4.3 ± 0.48	2.8 ± 0.26	2.1 ± 0.16	3.2 ± 0.11	2.7 ± 0.21	1.7 ± 0.09
	Middle	3.6 ± 0.23	3.9 ± 0.34	2.7 ± 0.19	0.9 ± 0.31	2.0 ± 0.21	2.6 ± 0.12	2.0 ± 0.06	0.8 ± 0.10
	End	1.7 ± 0.15	1.3 ± 0.11	1.8 ± 0.22	0.5 ± 0.09	1.1 ± 0.18	1.8 ± 0.12	1.4 ± 0.17	0.3 ± 0.08
**Itching sensation**	Beginning	3.2 ± 0.17	3.9 ± 0.38	2.7 ± 0.36	1.1 ± 0.20	2.7 ± 0.21	3.0 ± 0.17	3.1 ± 0.18	1.2 ± 0.12
	Middle	1.8 ± 0.13	2.2 ± 0.35	2.1 ± 0.28	0.5 ± 0.11	2.1 ± 0.18	1.7 ± 0.19	1.9 ± 0.16	1.0 ± 0.08
	End	2.1 ± 0.21	1.0 ± 0.09	1.2 ± 0.12	0.3 ± 0.10	1.5 ± 0.20	1.3 ± 0.06	0.9 ± 0.07	0.1 ± 0.03
**Burning sensation**	Beginning	-	-	-	-	-	-	-	-
	Middle	-	-	-	-	-	-	-	-
	End	-	-	-	-	-	-	-	-
**Not tolerated**	Beginning	-	-	-	-	-	-	-	-
	Middle	-	-	-	-	-	-	-	-
	End	-	-	-	-	-	-	-	-

The values are rated using Numeric Analogue Scale (NAS) 0 is rated as no sensation and 10 rated as the worst sensation imaginable. The sensations are recorded during three phases of stimulation: Beginning (0 to 7 minutes of stimulation), Middle (7 to 14 minutes of stimulation), End (14 to 20 minute of stimulation). Sensations under both active (anode) and reference (cathode) electrodes were recorded during a-tDCS of primary motor cortex (M1), primary sensory cortex (S1), dorsolateral prefrontal cortex (DLPFC), and sham tDCS. Scores are reported as mean ± SEM.

## Discussion

### The effects of a-tDCS of M1, S1, and DLPFC on M1 excitability

Our results indicate that there are significant differences between active and sham/no-tDCS in all conditions at T_0_ (except a-tDCS of S1), and at T_30_. Hence, we can conclude that our findings are due to the real effects of active a-tDCS, not placebo effects. M1 excitability increases by a-tDCS of both M1 and DLPFC, whereas a-tDCS of S1 produces an opposite effect immediately after intervention (at T_0_). Interestingly, the MEP sizes significantly increased 30 minutes after completion of S1 a-tDCS.

The findings in current study are in agreement with several other studies in which MEP increases after application of a-tDCS over the M1 [7, 35, 40, 43, 69]. Although compared to other studies, the electrode size and current intensity are different in our study; the current density is kept identical. Furthermore, as can be seen in Fig. 3, the M1 and DLPFC stimulation induced similar M1 corticospinal changes immediately after a-tDCS. Although there is no evidence showing the effects of a-tDCS of the DLPFC on M1 excitability yet, some anatomical studies suggest that the premotor cortex is divided into dorsal and ventral parts and the dorsal part sends its output to the M1 and spinal cord and receives prominent input from DLPFC [70, 71]. DLPFC activity is important during pain for maintenance of information in short-term memory and governing efficient performance control in the presence of painful stimuli by modulation of attention [72, 73]. The attention modulation signals from the DLPFC and motor preparation information from the dorsal part of the premotor cortex are received by the M1. These functional connections findings can explain why increasing the level of DLPFC stimulation leads to increased M1 excitability. As a result, based on the functional [74, 75] and anatomical [74, 76] relationship between DLPFC and M1, we conclude that a-tDCS of the DLPFC may activate the DLPFC-premotor-primary motor pathway and increase M1 excitability.

The mechanisms underlying these changes remain unclear. TDCS affects the stimulated area by a number of different mechanisms and is able to induce changes in different functional areas of brain [77]. TDCS induces physiological changes that result in local and distant plastic changes. Furthermore, the immediate after-effect of a-tDCS is associated with changes in neuronal membrane channels, such as sodium and calcium transporters [78–80]. After-effects of a-tDCS are also influenced by the potentiation of synaptic glutamatergic receptors [36].

After a-tDCS of S1, the level of M1 excitability remained unchanged immediately after stimulation and only increased significantly after a 30-minute delay. The cortico-cortical interconnections in superficial layers of the M1 and S1 are likely to be crucial for sensory input processing in S1 and sensorimotor integration [81–83]. It has been found that S1-projecting M1 pyramidal neurons strongly recruit a type of interneuron named Vasointestinal Peptide (VIP). VIP interneurons are inhibitory and fast-spiking. They account for the most *gamma*-Aminobutyric acid (GABAergic) interneurons in S1 and, are located in the superficial layers of S1 and target the distal dentrities of pyramidal cells in M1 [84–86]. A-tDCS of S1 might activate VIP interneurons, suppressing M1 excitability for a short period. It is likely that M1 excitability gradually increases after 30 minutes due to M1 and S1 projection [84] and due to short-term potentiation (STP) [87] and early long-term potentiation (e-LTP) [88] mechanisms. E-LTP depends on activation of calcium-dependent kinases, which control the trafficking of α-amino-3-hydroxy-5methyl-4isoxazolepropionic acid, and activation of N-methyl-D-aspartate—a subtype of glutamate receptor [89–91].

### The effects of a-tDCS of M1, S1, and DLPFC on S1 excitability

Our results show that there were significant differences between sham/no-tDCS and active a-tDCS of M1 and S1. Surprisingly, converse behavior was found following a-tDCS of M1 and S1; the N20-P25 component of the SEPs increased immediately after a-tDCS of S1, but decreased immediately after a-tDCS of M1.

In agreement with other SEP studies, application of a-tDCS over S1 increased the level of S1 excitability [20, 43]. In contrast with SEP studies in which it is reported that SEP facilitation lasted longer than one hour after 10 minutes of a-tDCS of S1 [20, 43], in our study 20 minutes of a-tDCS only increased the level of S1 excitability immediately after a-tDCS. Ion channel alteration in S1 is the probable mechanism behind the immediate effects of a-tDCS [78]. The current study is the first report of the effects of M1 and DLPFC stimulation on S1 excitability.

Few studies have investigated the integration of information between multiple cortical regions, including M1 and S1, in pain conditions [92, 93]. Some of these studies produced similar evidence of reduced MEP amplitude following S1 excitability reduction with experimental pain [92], whereas others failed to do so [94–96]. Schabrun et al. concluded that following pain induced S1 excitability reduction, M1 excitability and motor outputs also reduced, although there is no relationship between these measures. Schabrun et al. also indicated that S1 excitability reduction influences M1 processes, but the underlying mechanisms are not understood [92]. There are three possible explanations for the differences between the results; first, the individual variation is high [97], which may conceal or reveal excitability or perception changes in the studies. Second, different methodologies were used in the studies, which may influence the activities of different parts of the brain. Third, the active areas of the brain in experimental subjects experiencing pain are different to those in healthy subjects.

Suppression of N20-P25 amplitude after M1 stimulation may be explained by activation of the projections from motor to sensory cortex [6, 86]. These projections are mainly affect areas 1 and 2 of the sensory cortex. Any changes in these areas of S1 could be easily assessed by P25 and N33 amplitudes [98]. Moreover, some neuroimaging studies demonstrated that a-tDCS of M1 induces widespread bi-directional changes in regional neuronal activities, including thalamic nuclei [1]. Therefore any changes in the N20 component of SEP could be partially explained by activation of the sensory cortex by thalamo-cortical fibers [98]. Although, there is a possibility for contribution of cortico-subcortico-cortical reentry loops to the suppression of the sensory cortex, it is more likely that the observed suppression is produced by cortico-cortical effect from the motor cortex to sensory cortex.

### The effects of a-tDCS of M1, S1, and DLPFC on STh

In the current study, no significant changes were found between sham/no-tDCS conditions and the active ones. An immediate effect was observed after a-tDCS of M1. A significant increase was also observed 30 minutes after a-tDCS of M1 and DLPFC.

Our results are in line with those of Boggio et al. (2008), who concluded that a-tDCS of M1 but not DLPFC immediately increases STh [61]. Similarly, our recent meta-analysis showed that a-tDCS of both M1 and S1 increases STh immediately after a-tDCS [99]. However, we found that a-tDCS of DLPFC significantly increased STh after a 30 minute delay, while Boggio et al. found no STh increase [61]. This discrepancy may be explained by differences in methodologies in these studies, such as use of different electrode sizes (5×7cm by Boggio et al. vs. 1.5×2cm in our study), and current intensity (2mA vs. 0.3mA respectively) [46]. In addition, an a-tDCS study by Antal et al. demonstrated that both anodal and sham stimulations had no effect on STh [100]. Ragert et al. also found that a-tDCS of S1 enhances tactile spatial acuity rather than suppresses it [16].

Likewise, there is some evidence indicating that stimulation of M1, may increase the activity of insula and thalamus [101–103]. As a result, the insular-thalamus pathway activation following a-tDCS of M1 may be a possible explanation for the modulation of sensory/pain processing, leading to a STh increase.

### The effects of a-tDCS of M1, S1, and DLPFC on PTh

The significant difference between sham and no-tDCS conditions, and lack of significant difference between all three sites of stimulation and sham tDCS suggest that a-tDCS may have strong placebo effects on behavioural aspects of pain processing such as PTh. Compared to the no-tDCS condition, PTh significantly increased after 30 minutes of a-tDCS of M1, S1, DLPFC, as well as following sham tDCS.

Our results demonstrate that there is no difference between stimulation of superficial areas of PNM and a-tDCS of all three sites of stimulation increases PTh in healthy individuals. Therefore, it can be concluded that that PTh modulation with a-tDCS of M1, S1, and DLPFC may results in the inhibition of thalamic and brainstem nuclei activity, decreasing the hyperactivity in these areas that underlies chronic pain [61, 104]. Indeed, neuroimaging studies demonstrate that anodal M1 [1] and S1 [15] tDCS induces widespread bidirectional changes in regional neuronal activities, including thalamic nuclei. Therefore, in this study a-tDCS may be modulating thalamic inhibitory connections such that a bigger magnitude of stimulus is required to generate a perception response [102].

Our result revealed that a-tDCS at 0.3mA is associated with effective blinding when compared with the no-tDCS condition. Complete blinding is important in clinical studies with a subjective outcome measure [105], thus both assessor and participants were blinded in our study and it is highly likely that the sensory effects of active stimulation were similar to those of sham tDCS with electrode size of 1.5×2cm and amplitude of 0.3mA. The effects of sham and active tDCS on subjective measurements have been reported in other studies [12, 13, 106] and their results are in agreement with the results of current study. In contrast, some tDCS studies have shown no placebo effect; in these studies the electrodes were large (35cm^2^) and thus lacked the focal effect of small electrodes (3cm^2^ in the current study) [20], which possibly affected these studies’ results.

### The effects of a-tDCS of M1, S1, and DLPFC on P_p_Th

The results of applying a-tDCS over M1, S1, and DLPFC revealed no significant immediate effect on P_p_Th. A-tDCS of M1 and DLPFC resulted in a significant P_p_Th increase after 30 minutes. In addition, we found no significant difference between either active/sham tDCS or sham/no-tDCS conditions. Furthermore, no significant differences between sham and control or all three sites of stimulation and sham tDCS suggest that a-tDCS has placebo effects on mechanical pain processing. Our results are consistent with previous studies [32, 33] in demonstrating that a-tDCS of M1 and S1 has no significant effect on P_p_Th.

Mechanoreceptive inputs from large A-beta and small A-delta fibres are ended at the thalamic ventral caudal nucleus via dorsal column medial pathways [107]. As a result, it is possible that a bigger magnitude of stimulus is required to generate a mechanical pain perception [102].

Peripheral electrical stimulation recruits axons based on their diameter, starting with large-diameter fibers (Aß fibers) [108]; in contrast, mechanoreceptors excite myelinated large A-beta and small A-delta fibers and may be processed through anatomically different pathways. It seems that a-tDCS of M1 and DLPFC alters both pathways, resulting in PTh and P_p_Th increase after a-tDCS of M1 and DLPFC.

### Safety and side effects of a-tDCS

The findings of the current study suggest that the use of a-tDCS with small electrodes leads to minimal side effects in healthy individuals. The participants’ tolerance for a-tDCS with the small electrode size was compatible with that for the conventional electrode size at all sites of stimulation. No adverse effect such as seizure, headache, or nausea were recorded, and general discomfort (itching and/or tingling) was the most often reported side effect of both active and sham tDCS.

### Limitations of the study

Our findings must be interpreted in the context of several limitations. First, the data were obtained from a healthy population with no pain history; therefore the results may not necessarily be extrapolated to people with different types of pain. Second, the effects were evaluated in young participants (under 35 years); older individuals may respond differently to a-tDCS. Third, some studies have reported gender differences in responses to tDCS [109–111]; as most of our participants were women, it is possible that gender influenced our results. Fourth, the current study utilized a conventional electrode montage with the active electrode (anode) over target stimulation areas and the passive electrode (cathode) over contralateral supraorbital area (subgenual cortex). Therefore the findings in this study should be interpreted in light of the fact that all target stimulation sites received anodal stimulation and the subgenual cortex under the passive electrode received cathodal stimulation. This may become significant depending on the nature of the functional connectivity between the subgenual cortex and the PNM sites stimulated in this study.

### Suggestions for future research

Our study did not assess the effects of tDCS on M1 and S1 excitability beyond 30 minutes. Further studies are required to fully characterize the effects of tDCS over superficial PNM regions. The effects of a-tDCS application time, current intensity, and electrode size should be systematically studied to improve our understanding in this field. Furthermore, to reveal the mechanisms of action of a-tDCS of superficial PNM regions on the excitability of M1 and S1, M1 excitability should be studied by measuring silent period, intracortical inhibition and facilitation to indirectly assess the role of GABAa, GABAb and glutamergic receptors. Additional pharmacological experiments using receptor agonists/antagonists are needed to determine if a-tDCS of different areas of the PNM has different or similar mechanisms.

Further studies are also recommended to investigate the effects of cathodal tDCS of PNM regions on brain excitability and pain perception. In addition, the current study can be a pilot for further hypothesis generation regarding the complex mechanisms that are involved in response to brain stimulation. To increase homogeneity, controlling for hormonal status in female participants is recommended in future studies.

## Conclusion

In summary, the results of this study suggest that compared to a-tDCS of S1, a-tDCS of M1 and DLPFC are better techniques to enhance the excitability of M1. Furthermore, there is no site-specific effect on behavioral aspects of pain processing and a-tDCS of all superficial areas of PNM increased STh and PTh. Our findings can be employed to develop a-tDCS protocols for clinical applications of pain modulation. Our study provides valuable information about the best site of stimulation for future therapeutic strategies in neurorehabilitation and pain studies.

## Supporting Information

S1 CONSORT Checklist (DOC)

S1 Protocol Original protocol of study. (DOCX)
