# Supplementary material for: How Does Anodal Transcranial Direct Current Stimulation of the Pain Neuromatrix Affect Brain Excitability and Pain Perception? A Randomised, Double-Blind, Sham-Control Study
Source: PLoS One. 2015 Mar 4;10(3):e0118340. doi: 10.1371/journal.pone.0118340 (PMC4349802; doi:10.1371/journal.pone.0118340)
Supplement: S1 Protocol — (DOCX) [file pone.0118340.s002.docx]

**How does transcranial direct current stimulation of the pain neuromatrix affect brain excitability and pain perception? A randomised, double-blind, sham-control study**

1. **BACKGROUND**

Pain is the primary reason for patients to seek medical care. A survey found that 31% of the population in the united states and 19% of adult European population had experienced moderate to severe pain, with serious consequences for their social and working lives. The exorbitant medical treatment costs paid by governments and individuals can be decreased by better therapeutic approaches.

Parallel areas of brain are involved in experience of Pain which make it a complex issue to manage. Lateral parallel nuclei and somatosensory cortex (S1) are responsible for discrimination of quality, location, and intensity of pain, whereas medical thalamic nuclei, dorsolateral prefrontal cortex (DLPFC) (Nitsche et al. 2009; Nitsche et al. 2012), and limbic system have been proposed to subserve emotional dimensions of pain (Boros et al. 2008; Lang et al. 2005; Nitsche and Paulus 2000b). Furthermore, it is suggested that neural communications between S1 and primary motor cortex (M1) may leads to motor cortex neuroplastisity to reduce movement in order to prevent further injuries (Porro 2003).

Non-invasive therapeutic approaches, including medication, electrotherapy, and manual therapy, can provide satisfactory pain control in only 20%-30% of cases of pain syndromes. In order to develop a more effective treatment method, it is, therefore, needed to test other efficient methods. Transcranial direct current stimulation (tDCS), including anodal and cathodal tDCS, is one of the novel painless techniques used by neuroscientists to relieve pain (Boggio et al. 2006; Nitsche and Paulus 2000a). The optimal parameters and stimulation site, for effective application of tDCS, have not been studied yet. The findings of current study will be used to optimise the effects of new therapeutic approaches for pain relief.

1. **OBJECTIVE**

The primary aim is to simultaneously measure the level of M1 and S1 excitability following a-tDCS and c-tDCS of M1, S1, and DLPFC to investigate the functional connectivities between these sites in healthy individuals. The secondary aim was to investigate how M1 or S1 excitability modulation affects STh and PTh. We also aimed to investigate the placebo effects of a-tDCS on modulation of M1, S1 excitability and STh and PTh. Indeed, the results of this pilot study generate further hypotheses relating to complex mechanisms of different brain stimulation localisations on sensory/pain thresholds and may provide valuable information for future studies working on pain management.

1. **Methods**

The current study is a single-center, doubled-blinded, randomized, sham-controlled crossover study to determine the site-specific effect of a single session of a-tDCS on M1 and S1 excitability and STh and PTh in healthy volunteers.

**Intervention:**

In the current project, healthy participants will receive both a- and c-tDCS under each of eight different conditions in a random order: a-tDCS of M1 , a-tDCS of S1, a-tDCS of DLPFC, c-tDCS of M1, c-tDCS of S1, c-tDCS of DLPFC, sham, and no tDCS. The experimental sessions were separated by at least 72 hours to avoid interference or carry-over effects of tDCS, and completed at the same time of day to avoid diurnal variation. The duration of tDCS application was 20 minutes with amplitude of 0.3 mA and electrode size of 1.5 * 2 cm (current density of 0.029 mV/cm2) in all experiments.

**Outcome measures:**

The main outcome measures are the level of brain excitability in M1 and S1. To measure the level of excitability of M1, we will use Transcranial magnetic stimulation (TMS) on the M1 and record the EMG activity of First dorsi interossous (FDI) muscle. The peak to peak amplitude of motor evoked potentials recorded from FDI muscle indicates the level of primary motor cortex excitability.

To measure the level of excitability in S1, sensory evoked potential (SEP) will be measured and the peak to peak amplitude of N20-P25 responses generated in S1 will be measured.

Sensory (STh) and pain (PTh) threshold to electrical stimulation are the other outcome measures. Electrical stimulation will be applied by a pen electrode (model: 2762CC, Chattanooga, USA) to the right median nerve (pulse duration: 200 microsecond) at wrist level. Current supply will start at 0mA and will be increased in steps of 0.1mA until the participant reports sensation and pain. The intensity of current at which perception of the electrical stimulus is first reported will be

taken as the STh.

Pain threshold to the mechanical stimulation (PpTh)is the other outcome measure: Pressure was induced using a pressure algometer (model: FDX 50, Wagner, USA; capacity: 50× 0.05Ibf, accuracy: ±0.3% of full scale) with a flat circular metal probe dressed in a plastic cover. Force was displayed digitally in increments of 0.1N. The algometer was mounted vertically. For each measurement the algometer was calibrated to enable force to be applied at a controlled and steady rate. PpTh was defined as the amount of force required to elicit a sensation of pain distinct from pressure discomfort. The PpTh measurement point was marked in the middle of the belly of the FDI muscle.

All evaluations will be performed before (Tpre), immediately after (T0), 30 min (T30) and 60 min (T60) after the intervention. Also, one day after the intervention all evaluations will be repeated (Tday1).


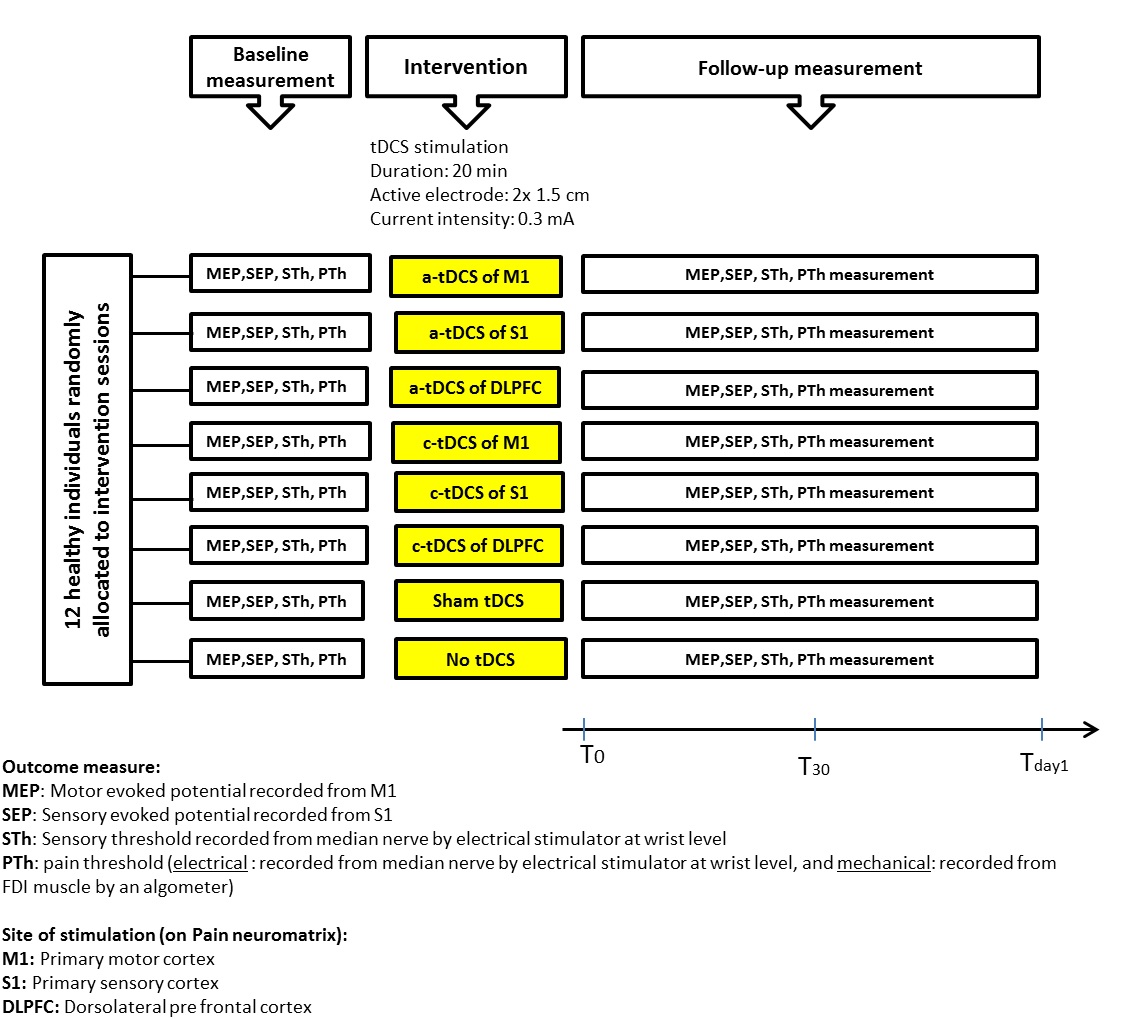


**Inclusion criteria:**

All participants should be:

- Healthy
- Right-handers as determined by the Edinburgh Handedness Inventory
- Between 18 and 49 years
- Males and females

**Exclusion criteria:**

Volunteers will be excluded if they have:

- Clinically significant or unstable medical, neuropsychiatric
- Chronic pain disorder
- History of substance abuse or dependence
- Any use of central nervous system-effective medication
- History of brain surgery, tumour, or intracranial metal implantation
- All participants will be interviewed and examined by a physician prior to enrolment in the study and provided written, informed consent.

**References:**

- Jeffery D T., Norton J A., Roy F D., Gorassini M A. (2007). Effects of transcranial direct current stimulation on the excitability of the leg motor cortex. *Exp Brain Res, 182*(2), 281-287..
- Lefaucheur, J. P., et al. (2012), 'Analgesic effects of repetitive transcranial magnetic stimulation of the motor cortex in neuropathic pain: Influence of theta burst stimulation priming', Eur J Pain, 16 (10), 1532-2149.
- Nitsche M A., Cohen L G., Wassermann E M., Priori A., Lang N., Antal A., et al. (2008). Transcranial direct current stimulation: State of the art. *Brain Stimul, 1*(3), 206-223.
- Nitsche M A., Nitsche M S., Klein C C., Tergau F., Rothwell J C., Paulus W. (2003). Level of action of cathodal DC polarisation induced inhibition of the human motor cortex. *J Clin Neurophysiol, 114*(4), 600-604.
- Nitsche, M. A. and Paulus, W. (2000), 'Excitability changes induced in the human motor cortex by weak transcranial direct current stimulation', J Physiol, 527 Pt 3, 633-9.
- Nitsche, M. A., et al. (2008), 'Transcranial direct current stimulation: State of the art 2008', Brain Stimul, 1 (3), 206-23.
- Nitsche, M. A., et al. (2012), 'Effects of frontal transcranial direct current stimulation on emotional state and processing in healthy humans', Front Psychiatry, 3 (58), 18.
- Pascual-Leone A., Valls-Sole J., Wassermann E M., Hallett M. (1994). Responses to rapid-rate transcranial magnetic stimulation of the human motor cortex. *Brain, 117*(Pt 4), 847-858.
- Porro, C. A. (2003). "Functional imaging and pain: behavior, perception, and modulation." Neuroscientist **9**(5): 354-369.
- Wang, W., J. L. Collinger, et al. (2010). "Neural interface technology for rehabilitation: exploiting and promoting neuroplasticity." Phys Med Rehabil Clin N Am **21**(1): 157-178.
- Webster, BR, Celnik, PA, and Cohen, LG (2006), 'Noninvasive brain stimulation in stroke rehabilitation', NeuroRx, 3 (4), 474-81.
